# Bisphenol A Exposure Alters Developmental Gene Expression in the Fetal Rhesus Macaque Uterus

**DOI:** 10.1371/journal.pone.0085894

**Published:** 2014-01-23

**Authors:** Kathryn C. Calhoun, Elizabeth Padilla-Banks, Wendy N. Jefferson, Liwen Liu, Kevin E. Gerrish, Steven L. Young, Charles E. Wood, Patricia A. Hunt, Catherine A. VandeVoort, Carmen J. Williams

**Affiliations:** 1 Reproductive Medicine Group, Laboratory of Reproductive & Developmental Toxicology, National Institute of Environmental Health Sciences, Research Triangle Park, North Carolina, United States of America; 2 Department of Obstetrics and Gynecology, Division of Reproductive Endocrinology and Infertility, University of North Carolina-Chapel Hill, Chapel Hill, North Carolina, United States of America; 3 Microarray Group, National Institute of Environmental Health Sciences, Research Triangle Park, North Carolina, United States of America; 4 National Health and Environmental Effects Research Laboratory, Office of Research and Development, US Environmental Protection Agency, Research Triangle Park, North Carolina, United States of America; 5 School of Molecular Biosciences, Washington State University, Pullman, Washington, United States of America; 6 Department of Obstetrics and Gynecology and California National Primate Research Center, University of California Davis, Davis, California, United States of America; Baylor College of Medicine, United States of America

## Abstract

Bisphenol A (BPA) exposure results in numerous developmental and functional abnormalities in reproductive organs in rodent models, but limited data are available regarding BPA effects in the primate uterus. To determine if maternal oral BPA exposure affects fetal uterine development in a non-human primate model, pregnant rhesus macaques carrying female fetuses were exposed orally to 400 µg/kg BPA or vehicle control daily from gestation day (GD) 50–100 or GD100–165. Fetal uteri were collected at the completion of treatment (GD100 or GD165); tissue histology, cell proliferation, and expression of estrogen receptor alpha (ERα) and progesterone receptor (PR) were compared to that of controls. Gene expression analysis was conducted using rhesus macaque microarrays. There were no significant differences in histology or in the percentage of cells expressing the proliferation marker Ki-67, ERα, or PR in BPA-exposed uteri compared to controls at GD100 or GD165. Minimal differences in gene expression were observed between BPA-exposed and control GD100 uteri. However, at GD165, BPA-exposed uteri had significant differences in gene expression compared to controls. Several of the altered genes, including *HOXA13*, *WNT4*, and *WNT5A*, are critical for reproductive organ development and/or adult function. We conclude that second or third trimester BPA exposure does not significantly affect fetal uterus development based on morphological, proliferation, and steroid hormone receptor assessments. However, differences in expression of key developmental genes after third trimester exposure suggest that BPA could alter transcriptional signals influencing uterine function later in life.

## Introduction

The disruptive effects of inappropriate estrogenic chemical exposure on human female reproductive tract development are well established. A dramatic example is provided by the potent synthetic estrogen diethylstilbestrol, which leads to numerous structural and functional reproductive tract abnormalities as a consequence of prenatal exposure [1]. Although diethylstilbestrol is no longer used in pregnant women, there are concerns that environmental exposures to other estrogenic endocrine disrupting compounds could affect fetal reproductive tract development. Bisphenol A (BPA) is a widely used industrial chemical with estrogenic activity. As a component of polycarbonate plastics and epoxy resins, BPA is incorporated into food containers, cans, dental sealants, and thermal receipts. It leaches out of these parent compounds with heating and degradation and exposure is nearly ubiquitous in industrialized countries [2], [3], [4].

BPA binds nuclear estrogen receptors α (ERα) and β to modulate transcriptional activity, influences non-genomic estrogen-mediated signaling, and may affect both thyroid hormone and glucocorticoid signaling (reviewed in [3]). In animal models, BPA is an endocrine disrupting compound with regard to development and function of numerous tissues including the brain, mammary gland, ovary, and reproductive tract [3], [5]. Developmental exposure of rodents to BPA has lifelong consequences for reproductive function, including alterations in timing of puberty, altered estrous cyclicity, and increases in the expression of ERα and progesterone receptor (PR) in the uterus [3], [5]. Effects of BPA vary by stage of life at time of exposure, and differences in metabolism and tissue sensitivity may make the fetus more vulnerable to lower levels of BPA exposure [6]. In women, BPA levels in amniotic fluid are markedly higher than in maternal plasma, suggesting that the human fetus may have significant exposures during development [7].

There is little definitive information about the human health effects of BPA. Some epidemiologic studies suggest that BPA exposure may be associated with adverse health effects, including developmental outcomes; however, most of these studies have been case-control, cross-sectional, retrospective, and/or limited in size (reviewed in [4]). To help address concerns over human BPA exposure early in life, in this study a rhesus macaque animal model was used to examine the effects of prenatal exposure to BPA on the development of several tissues shown previously in rodent models to be affected by BPA. This model was used because rhesus macaques (*Macaca mulatta*) are similar to humans with regard to BPA metabolism, physiology and pharmacology [8] and share many features of reproductive organ physiology and development [9], [10]. Results for three of the tissues from this study, ovary, mammary gland, and brain, have been published [11], [12], [13]. The current report describes changes in fetal uterine histology and gene expression from mid to late gestation and the associated effects of oral maternal BPA exposure.

## Materials and Methods

### Ethics Statement

All animal protocols were approved prior to implementation by the Institutional Animal Care and Use Committee (IACUC) at the University of California, Davis, and all procedures conformed to the requirements of the Animal Welfare Act. Activities related to animal care including housing, feeding, and environmental enrichment were performed in accordance with IACUC-approved standard operating procedures (SOPs) at the California National

Primate Research Center (http://www.cnprc.ucdavis.edu). Briefly, animals were individually housed in stainless steel cages under a 12∶12 hr light-dark cycle at 25–27°C. They were fed Purina Monkey Chow and water ad lib; environmental enrichments included cereal, seeds, and seasonal produce. Fetuses were euthanized immediately after delivery and newborns within 24 hours of delivery in a manner consistent with the recommendations of the American Veterinary Medical Association (AVMA) Guidelines on Euthanasia and Primate Center SOPs (IV administration of pentobarbital overdose).

### Animals and treatments

The rhesus macaques used in these studies were the same cohort of macaques that received oral treatments described previously [11], [12], [13]. Briefly, healthy pregnant females carrying female fetuses were randomly allocated to one of two treatment groups that received a daily fruit treat containing either 400 µg/kg deuterated BPA (CDN Isotopes, Quebec, Canada) or vehicle (100 µl ethanol). The macaques were subdivided into two groups based on gestational timing. The “GD100” group (N = 5 BPA-treated, N = 5 control) was treated from gestational day (GD) 50–100; delivery was on GD100 by caesarian section. The “GD165” group (N = 6 BPA-treated, N = 6 control) was treated from GD100 until vaginal delivery at approximately GD165. After delivery, the fetal uterus was removed and cut sagitally from fundus to cervix; one side was fixed for histological evaluation, and the other half was frozen for analysis of gene expression. There were five idiopathic stillbirths (N = 2 control, N = 3 BPA-exposed) in the GD165 treatment group. Uteri from stillborn fetuses were not included in the microarray analyses.

### Total RNA isolation, microarray analysis, and real-time RT-PCR

Total RNA was isolated using the RNeasy Mini kit (Qiagen, Valencia, CA). Gene expression analysis was conducted on RNA from individual uterus samples using rhesus macaque gene expression microarrays (V1:015421; Agilent Technologies, Santa Clara, CA) following the Agilent 1-color analysis protocol. Data were obtained using Agilent Feature Extraction software (v9.5; 1-color defaults), which performed error modeling, adjusting for additive and multiplicative noise. An error-weighted ANOVA and Benjamini-Hochberg multiple test correction with p-value<0.01 was performed using Rosetta Resolver® (Rosetta Biosoftware, Kirkland, WA) to identify differentially expressed probes. Additional analyses were performed using Partek Genomics Suite (Partek Inc., St. Louis, MO) to create principal component analyses and hierarchical clustering plots. Pathway and network-based analyses were performed on differentially expressed probes using IPA 9.0 (Ingenuity Systems, Redwood City, CA). Analyses were performed for each comparison on a dataset restricted to differentially expressed probes having a 2.0-fold (controls, GD100 vs. GD165) or a 1.3-fold (GD165; BPA vs. control) change cutoff to achieve suitable numbers of probes for the analyses. The microarray data were deposited in the National Center for Biotechnology Information Gene Expression Omnibus, accession no. GSE38421.

For real time RT-PCR, first strand cDNA was generated from 1 µg total RNA. Primers were designed across exon junctions using Primer3Web 0.4.0 [14] based on rhesus macaque sequences (Table S1). Real time RT-PCR was performed on 20 ng cDNA using SYBR® Green–based detection on an ABI 7200 HT detector (Applied Biosystems, Foster City, CA) as described previously [15]. Relative gene expression was calculated using the ΔΔC_t_ method with cyclophilin A (*Ppia*) expression for normalization [16].

### Histology and immunohistochemistry

Fixed uterine tissues were paraffin embedded, sectioned at 6 µm, stained with hematoxylin and eosin and examined by a board-certified pathologist for histologic changes. For immunohistochemistry, epitope retrieval was performed using 10 mM citrate buffer, pH 6.0 in a pressure cooker. Endogenous peroxidase was quenched with 3% hydrogen peroxide and sections were blocked using 10% horse serum followed by avidin/biotin blocking reagent (Vector Laboratories, Burlingame, CA). The following primary monoclonal antibodies and dilutions were used: anti-ER (1∶100; clone ER1D5; Beckman Coulter, Brea, CA); anti-Ki-67 (1∶100; clone MIB-1; DakoCytomation, Carpenteria, CA); anti-PR (1∶50; GeneTex, Irvine, CA), and anti-beta-catenin (1∶100; BD Transduction Laboratories, San Jose, CA). For all antibodies, the same dilution of mouse IgG_1_ (BD Pharmingen, San Diego, CA) was used as an isotype control. Secondary antibody was from the Vectastain Elite kit (Vector Laboratories) and diaminobenzamine was used as the chromogen. Sections were counterstained with hematoxylin.

### Quantitation of immunostaining

Slides were scanned and viewed using the Aperio® Scanscope XT Digital Slide Scanner and ImageScope software (Aperio Technologies, Vista, CA). Five regions per slide of luminal epithelium 100 microns in length were selected near the uterine fundus for epithelial cell counts. Five regions per slide of 100 square µm were selected for stromal cell counts. Image analysis was performed by an observer blinded to treatment status using Aperio® IHC Nuclear Algorithm v. 8.001, which measures the area, number of positive-stained nuclei, number of counter-stained nuclei, and the total number of nuclei. For each animal, the percentage of positive nuclei was calculated as the average of the five field measurements in the epithelial or stromal regions. The data were analyzed with Prism 6.0 software (GraphPad, La Jolla, CA) using the Kruskal-Wallis test and Dunn's multiple comparison test with a significance level set at p<0.05. Because there was no difference in the percentage of positive nuclei in the epithelial and stromal compartments for any of the antibodies, the data were combined for presentation.

## Results

The dams included in this study were given an oral dose of BPA that resulted in average serum concentrations of unconjugated biologically active BPA of ∼0.3–0.5 ng/ml [12], which is similar to levels found in human adults and fetuses [2], [3]. Control macaques had negligible serum BPA concentrations. There were no significant differences between treatment groups in the body weights of the mothers or fetuses at the time of delivery [12].

### Histological analysis

**Figure 1:**
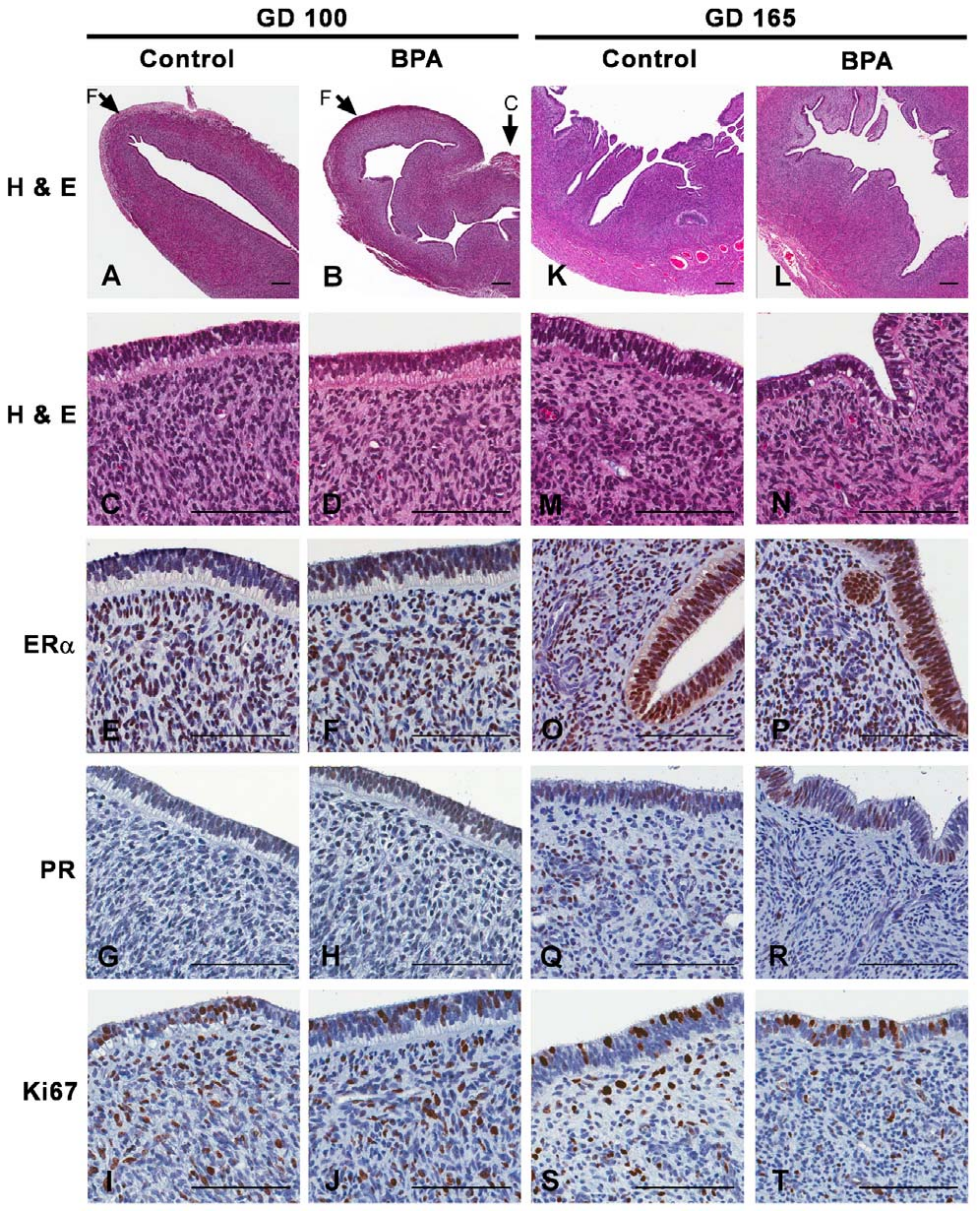
Histology and ERα, PR, and Ki-67 immunolabeling of uteri from control and BPA-treated macaques at GD100 and GD165. Treatment group and staining method or antibody are indicated. In panels A and B, “F” indicates uterine fundus and “C” indicates uterine cervix. Scale bars = 100 µm.

Uteri from GD100 fetuses had well-developed myometrium, endometrial stroma and luminal columnar epithelium (Fig. 1A–D). Bud formation and rudimentary tubules were evident within the superficial endometrial epithelium, but no tubular branching or mature endometrial glands were observed (Table 1). No gross or histologic uterine lesions were identified. Minimal to marked cytoplasmic vacuolization, predominantly subnuclear in location, was present in uterine epithelial cells in all animals, similar to that reported previously in cynomolgus macaques (*Macaca fascicularis*) [9]. There were no differences in the gross or microscopic appearance of the uteri between control and BPA-exposed animals, including blinded qualitative assessments of epithelial cell height and extent of subnuclear vacuolization. There were also no significant differences in the cellular distribution or nuclear staining patterns for ERα, PR, or the proliferation marker Ki-67 between GD100 control and BPA-exposure groups (Fig. 1E–J).

**Table 1:** Summary of histological findings in the uterus.

Microscopic Findings	GD100 control	GD100 BPA	GD165 control	GD165 BPA
Number of animals examined	5	5	6	6
No tubulogenesis observed (# animals)	2	0	0	0
Bud formation (# animals)	3	5	6	6
Rudimentary tubulogenesis (# animals)	2	2	6	6
Branching (# animals)	0	0	6	6
Extent of tubulogenesis grade^a^ (range)	0–2	1–2	4–5	4–5
Extent of tubulogenesis grade^a^ (average ± SE)	1.0±0.4	1.4±0.2	4.2±0.2	4.7±0.2
Luminal epithelial cell height (µm; range)	24.5–38.7	20.4–40.4	23.6–32.1	27.2–31.8
Luminal epithelial cell height (µm; average ± SE)	30.5±2.9	30.4±3.5	28.7±1.3	29.4±0.8
Subnuclear vacuolization grade^b^ (range)	1–4	1–3	1–5	1–4
Subnuclear vacuolization grade^b^ (average ± SE)	2.6±0.5	2.4±0.4	2.3±0.6	2.5±0.7

a Grades for extent of tubulogenesis: 0 = none present; 1 = bud formation only; 2 = buds and initial tubules, no branching; 3 = tubules predominant, no branching; 4 = tubules predominant, a few branches present; 5 = branching prominent.

b Grades for extent of epithelial subnuclear vacuolization: 0 = No vacuolization present; 1 = Minimal vacuolization (≤10% of cells); 2 = Mild vacuolization (11–25% of cells); 3 = Moderate vacuolization (26–50% of cells); 4 = Marked vacuolization (51–75% of cells); 5 = Severe vacuolization (>75% of cells).

Uteri from GD165 fetuses also showed well-developed myometrial and endometrial stromal and columnar epithelial layers (Fig. 1K–N). The major difference at this gestational age was more advanced glandular morphogenesis indicated by the presence of increased luminal epithelial invaginations and tubular epithelial structures within the endometrial stroma; tubular branching was observed in all GD165 uteri. Tubular development, epithelial cell height, and subnuclear vacuolization scores did not differ between treatment groups. Nuclear ERα, PR, and Ki-67 staining was present in both epithelial and stromal compartments (Fig. 1O–T).

**Figure 2:**
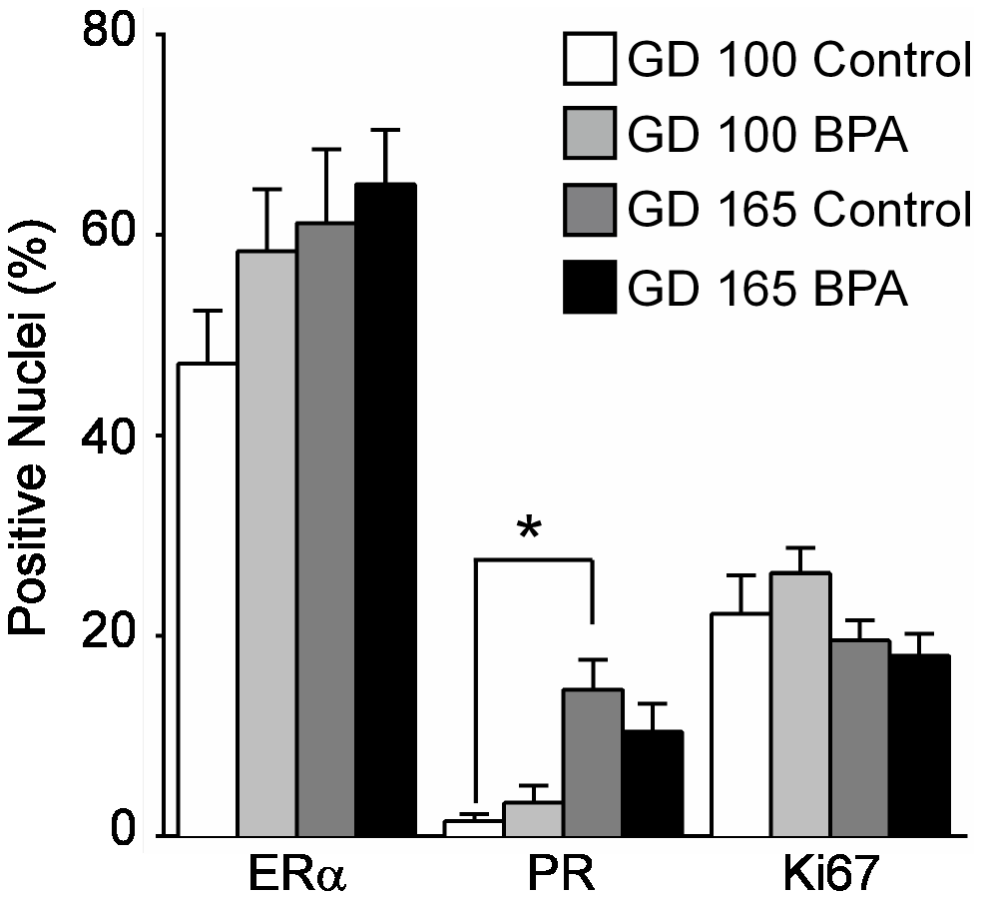
Comparison of ERα, PR, and Ki-67 labeling indices at GD100 and GD165. Graph indicates mean ± SEM of combined epithelial and stromal staining for each antibody. Single asterisk, p<0.05.

Quantitative analysis demonstrated no significant differences in the percentage of cells with positive staining for ERα, PR, or Ki-67 when comparing BPA-exposed to control uteri at either developmental time point (Fig. 2). Staining for ERα was present in ∼50% of nuclei in both epithelial and stromal compartments at both time points. Less than 5% of nuclei in either compartment stained for PR at GD100. In controls animals, PR staining increased to ∼15% at GD165; the increase in PR staining in BPA-exposed animals did not reach statistical significance. About 20% of the cells in all groups showed evidence of proliferative activity based on positive Ki-67 staining. At both GD100 and GD165, significantly fewer cells were positive for PR compared to ERα across both treatment groups (Fig. 2).

### Microarray analysis: Comparison of GD100 to GD165 control animals

**Figure 3:**
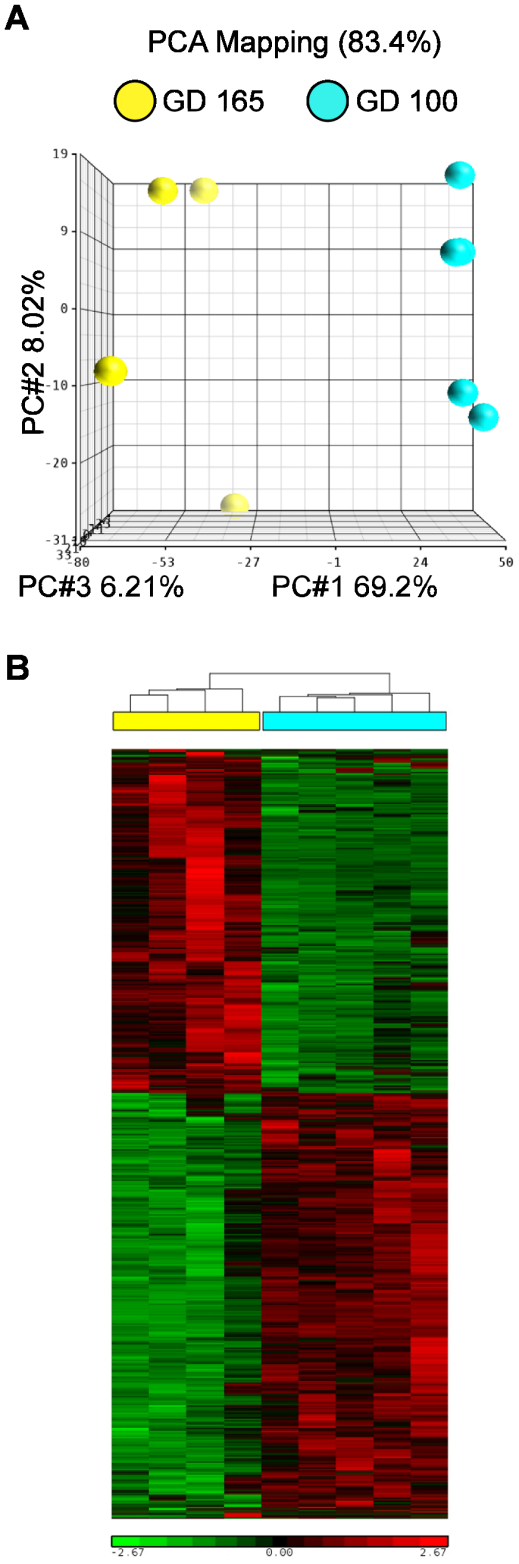
Microarray analysis of uteri from control macaques at GD100 and GD165. A. Principal component analysis. B. Hierarchical analysis. GD100 marked in cyan, GD165 marked in yellow. BPA treatment group marked in cyan; control group marked in yellow. Scale bars represent standardized signal intensity.

Uterine gene expression profiles showed marked differences in control animals based on developmental stage. Principal component analysis demonstrated clear separation between GD100 and GD165 animals (Fig. 3A). Of the 20,217 probes on the array, 3645 probes were significantly different after correction for multiple testing; of these, 406 were altered at least 2-fold. Hierarchical clustering was performed after filtering the data for significantly different probes; this analysis indicated clear differences in gene expression between uteri of macaques at different gestational ages and low inter-individual variability within each control group (Fig. 3B). A list of the most highly up- and down-regulated genes was generated (Table 2). Both up- and down-regulated probes from the microarray expression data were validated by real time RT-PCR (Table S1).

**Table 2:** Most highly up- and down-regulated genes in uteri from GD165 compared to GD100^a^.

Gene	Gene description	Microarray Fold Change (GD165/GD100)	p-value
*HBB*	hemoglobin, beta	61.74	9.28E-08
*CKM*	creatine kinase M-type (Creatine kinase, M chain) (M-CK)	33.71	6.74E-03
*CLDN10*	claudin 10	21.96	3.27E-06
*FAM180A*	family with sequence similarity 180, member A	19.77	1.00E-05
*MYH8*	myosin, heavy chain 8, skeletal muscle, perinatal	19.27	4.64E-08
*SLN*	sarcolipin	18.39	1.90E-04
*KLF9*	Kruppel-like factor 9	17.27	9.30E-15
*MYL1*	myosin, light chain 1, alkali; skeletal, fast	16.74	1.09E-03
*CAPN13*	calpain 13	16.20	1.18E-03
*MYH2*	myosin, heavy chain 2, skeletal muscle, adult	14.25	1.57E-09
*FDCSP*	follicular dendritic cell secreted protein	−5.67	9.30E-15
*OIT3*	oncoprotein induced transcript 3	−5.82	2.70E-13
*CSMD3*	CUB and Sushi multiple domains 3	−5.87	2.80E-08
*TEX15*	testis expressed 15	−6.38	7.82E-09
*GABRA1*	gamma-aminobutyric acid (GABA) A receptor, alpha 1	−7.78	4.05E-03
*LRRTM3*	leucine rich repeat transmembrane neuronal 3	−7.94	2.60E-04
*CRYGC*	crystallin, gamma C	−7.94	1.83E-12
*CABS1*	calcium-binding protein, spermatid-specific 1	−8.14	7.27E-10
*PCDH8*	protocadherin 8	−9.86	1.30E-04
*AMBN*	ameloblastin (enamel matrix protein)	−54.78	1.05E-13

a List filtered to keep only the genes with an average microarray intensity value ≥50 in either group.

**Figure 4:**
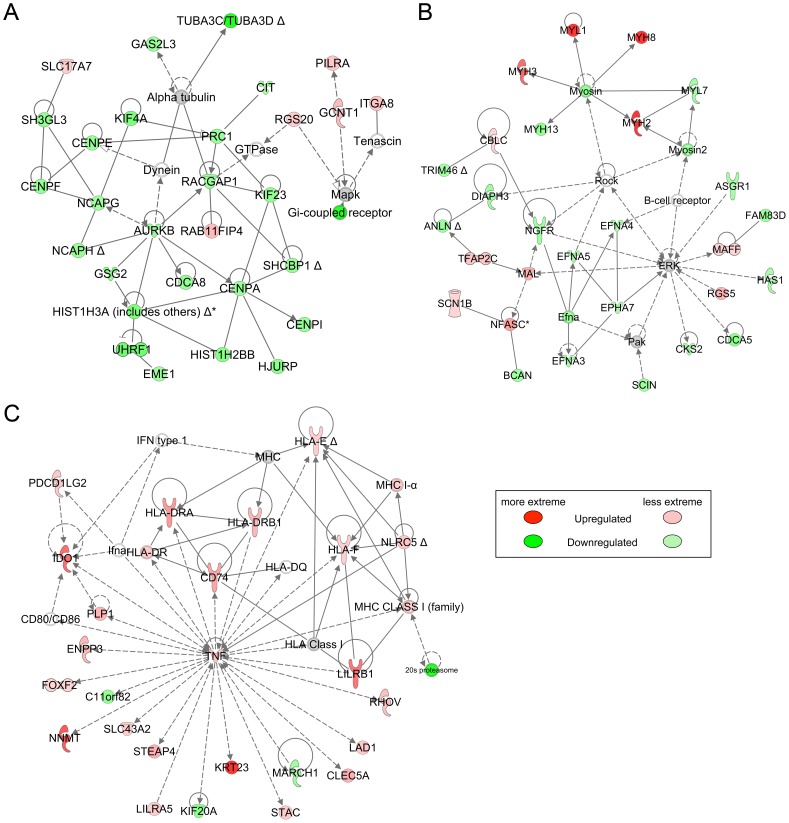
Top Ingenuity networks identified in Control GD100 vs. GD165 comparison. A. Cell Cycle, Cellular Assembly and Organization, DNA Replication, Recombination, and Repair (score = 40, 29 focus molecules, p value of top functions = 2.78E-08). B. Developmental Disorder, Neurological Disease, Organismal Injury and Abnormalities (score = 38, 28 focus molecules, p value of top functions = 2.09E-04). C. Inflammatory, Neurological, and Immunological Diseases (score = 32, 25 focus molecules, p value of top functions = 1.71E-05).

Ingenuity Core analysis identified several gene networks with significant alterations. The biological functions in the highest scoring network related to the cell cycle and cell division, including aurora kinase B, several centromere proteins, and alpha tubulin isoforms; most of the genes in this network were downregulated (Fig. 4A). The second network included a hub with several very highly upregulated myosin genes (Fig. 4B). A third network of note centered almost completely on tumor necrosis factor (*TNF*) as a hub (Fig. 4C). *TNF* itself was upregulated almost 3-fold, and the majority of the remaining genes, including many major histocompatibility complex (MHC) genes, were upregulated at least that much.

The biological functions for which the predicted activation state was significantly increased or decreased in the Ingenuity analysis are shown in Table S2. Based on the corresponding annotations in this table, the vast majority of the increased activation state functions can be explained by increases in hematopoietic cells in the uterus late in gestation. This finding is consistent with hemoglobin B being by far the most highly upregulated gene in the GD165 group (Table 2) and also with the upregulation of a large number of MHC genes in the *TNF*-centered network (Fig. 4C). The decreased activation state functions mainly related to the cell cycle, and are reflected in the downregulation of cell cycle genes shown in the top-scoring network (Fig. 4A). The upstream regulators identified by the Ingenuity analysis included cell cycle-related genes that were mainly downregulated if they generally function to promote cell division, e.g., *CCND1* and the *E2F* genes, and upregulated if they inhibit cell cycle progression, e.g., *RB1*, *CEBPA*, and *TP53* (Table S3). Indeed, one of the most highly upregulated genes, Kruppel-like factor 9 (*KLF9*) (Table 2), encodes a PR-interacting protein that modulates expression of genes important in cell cycle control [17].

### Microarray analysis: Comparison of BPA-exposed to control animals

Gene expression differences between uteri of the GD100 BPA-exposed and control fetuses were minimal. Principal component analysis of these two groups demonstrated very little segregation by treatment; this finding was confirmed by hierarchical cluster analysis (data not shown). There were only 84 differentially expressed genes identified; of these, only 5 were altered more than 2-fold. Based on these observations, further comparison of the microarray data between these groups was not carried out.

**Figure 5:**
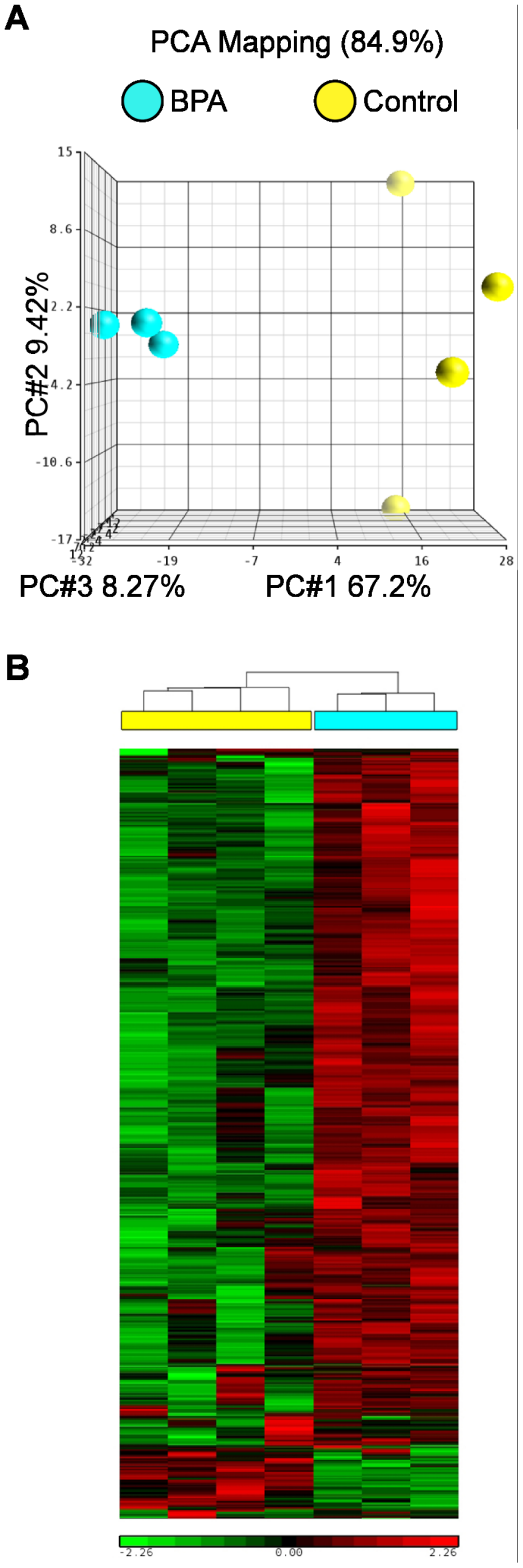
Microarray analysis of uteri from control and BPA-treated macaques at GD165. A. Principal component analysis. B. Hierarchical analysis. BPA treatment group marked in cyan; control group marked in yellow. Scale bars represent standardized signal intensity.

In contrast to the findings at GD100, there were significant differences in gene expression when comparing the GD165 control and BPA-exposure groups. Principal component analysis demonstrated segregation by treatment group (Fig. 5A). There were 883 significantly different probes and, of these, 179 were altered at least 1.5-fold. Hierarchical clustering was performed after filtering the data for significantly different probes; this analysis indicated clear differences in gene expression and good consistency within the treatment groups (Fig. 5B). Both up- and down-regulated probes from the microarray expression data were validated by real time RT-PCR (Table S1). A list of the most highly up- and down-regulated genes was generated (Table 3).

**Table 3:** Most highly up- and down-regulated genes in response to late gestation BPA treatment (GD165)^a^.

Gene	Gene description	Microarray Fold Change (BPA/Control)	p-value^b^
*PDE11A*	phosphodiesterase 11A	2.79	6.99E-09
*HOXC9*	homeobox C9	2.51	1.30E-11
*IGHMBP2*	immunoglobulin mu binding protein 2	2.50	2.17E-10
*CSTL1*	cystatin-like 1 precursor	2.50	2.10E-04
*HOXC10*	homeobox C10	2.46	6.95E-03
*IL26*	interleukin 26	2.37	1.54E-13
*KLK3*	kallikrein-related peptidase 3	2.31	1.54E-13
*ALX3*	ALX homeobox 3	2.25	1.00E-05
*DOK6*	docking protein 6	2.22	5.00E-05
*ABHD1*	abhydrolase domain containing 1	2.17	2.20E-04
*CDH4*	cadherin 4, type 1, R-cadherin (retinal)	−2.79	4.31E-03
*GDE1*	glycerophosphodiester phosphodiesterase 1	−2.85	5.84E-09
*GJB3*	gap junction protein, beta 3, 31kDa	−2.86	6.53E-06
*TFAP2C*	transcription factor AP-2 gamma (activating enhancer binding protein 2 gamma)	−3.06	3.80E-04
*RNF186*	ring finger protein 186	−3.12	8.40E-04
*HOXA13*	homeobox A13	−3.13	1.80E-04
*FGF10*	fibroblast growth factor 10	−3.40	1.51E-11
*CLIC6*	chloride intracellular channel 6	−4.89	2.46E-08
*CXCL14*	chemokine (C-X-C motif) ligand 14	−5.32	1.30E-04
*SST*	somatostatin	−9.05	8.33E-03

a List filtered to keep only the genes with an average microarray intensity value ≥50 in either group.

Based on work in mice and women, homeobox transcription factors encoded by *Hox* genes and secreted signaling proteins encoded by *Wnt* family genes have important roles in female reproductive tract development [18]. Several *HOX* and *WNT* genes and *FZD2*, which encodes a WNT receptor, were among those identified by microarray analysis to be differentially regulated in response to BPA exposure in the GD165 gestation group, although the fold changes observed were not high (Table 4). In addition, expression levels were well above background for *HOXB3*, several *HOXC* and *HOXD* genes, and *WNT2*, suggesting that there may be as yet undefined roles for *HOX* and *WNT* paralogs in primate reproductive tract development.

**Table 4:** List of altered homeobox and *Wnt/Fzd* signaling genes in response to late gestation BPA treatment (GD165)^a^.

Gene	Gene description	Microarray Fold Change (BPA/Control)	p-value	Contol Intensity	BPA Intensity
*HOXA13*	homeobox A13	−3.13	1.80E-04	1865.09	536.40
*HOXC6*	homeobox C6	1.74	3.00E-04	1476.25	3040.11
*HOXC8*	homeobox C8	1.73	7.90E-04	186.02	350.29
*HOXC9*	homeobox C9	2.51	1.30E-11	268.87	758.71
*HOXC10*	homeobox C10	2.46	6.95E-03	29.32	75.50
*HOXD1*	homeobox D1	1.48	3.85E-03	321.27	500.31
*HOXD3*	homeobox D3	1.83	3.50E-04	657.45	1349.09
*HOXD9*	homeobox D9	1.30	9.84E-03	6344.27	9241.96
*WNT2*	Wingless-type MMTV integration site family member 2	1.99	4.88E-03	426.42	1077.09
*WNT4*	Wingless-type MMTV integration site family member 4	1.56	6.00E-05	2650.91	4661.60
*WNT5A*	Wingless-type MMTV integration site family member 5a	1.28	1.00E-05	12231.18	17578.10
*FZD2*	frizzled homolog 2 (Drosophila)	1.14	2.40E-04	6136.87	7883.45

a List filtered to keep only the genes with an average microarray intensity value ≥50 in either group.

**Figure 6:**
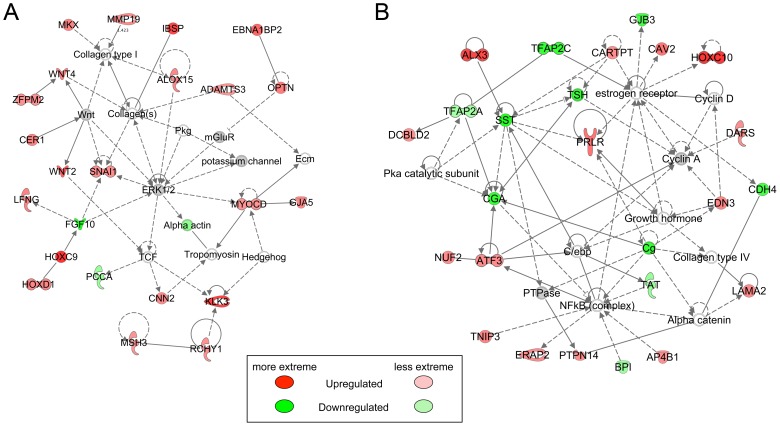
Top Ingenuity networks identified in GD165 Control v. BPA comparison. A. Post-Translational Modification, Protein Degradation, Protein Synthesis (score = 37, 20 focus molecules, p value of top functions = 7.21E-05). B. Cellular, Embryonic, and Organ Development (score = 37, 20 focus molecules, p value of top functions = 6.53E-05).

**Figure 7:**
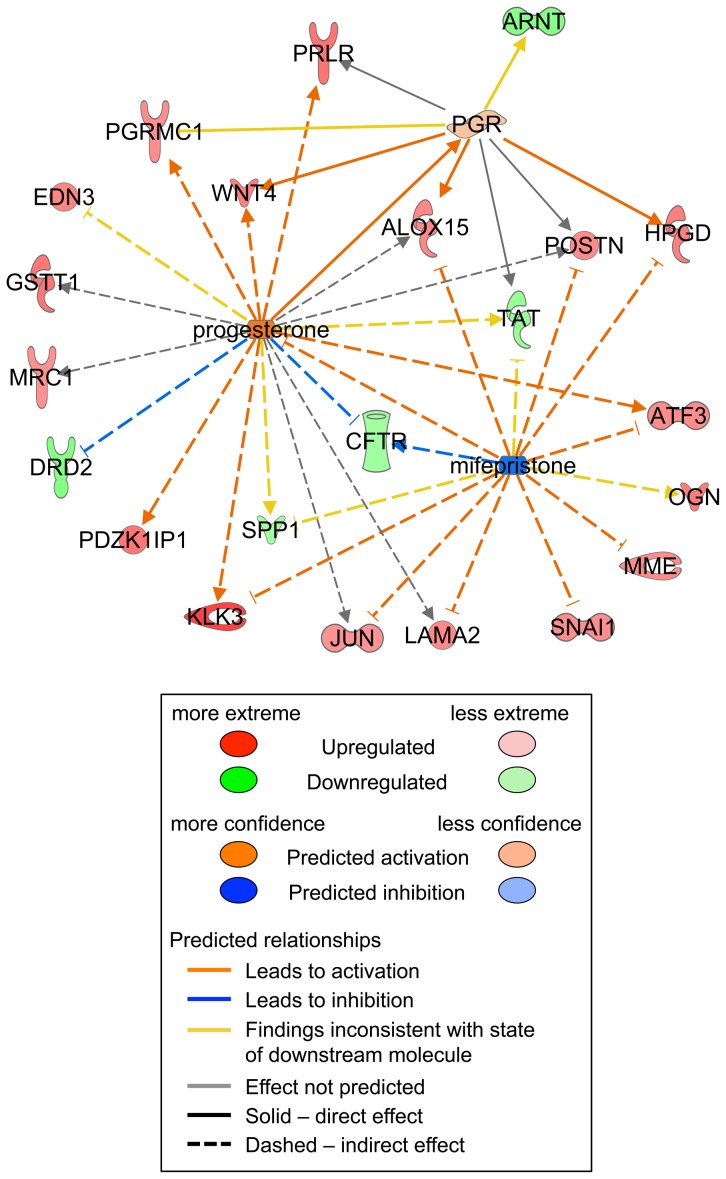
Alterations in progesterone- and mifepristone-regulated upstream regulatory networks in uteri from control and BPA-treated macaques at GD165. For progesterone, activation z-score = 1.53, p-value of overlap 1.59E-03. For mifepristone, activation z-score = −1.88, p-value of overlap 4.13E-04. Solid lines, direct interaction; Dashed lines, indirect interaction.

Two non-overlapping networks generated by the Ingenuity Core analysis stood out as highly significant. The top functions for the first of these networks included Post-Translational Modification, Protein Degradation, and Protein Synthesis; this network included several *HOX* and *WNT* genes (Fig. 6A). Top functions for the second network included Cellular and Embryonic Development; this network utilized the estrogen receptor as a hub (Fig. 6B). Differentially expressed molecules were linked by the Ingenuity analysis into mechanistic networks determined by upstream regulators. Potential upstream regulators included steroid hormones or regulators of steroid hormone signaling, including mifepristone, progesterone, androgen receptor, and dihydrotestosterone (Table S4). The molecules comprising the mechanistic network regulated by progesterone included the prolactin receptor and progesterone receptor membrane component 1, which are positively regulated by progesterone and were expressed more highly in BPA-exposed animals than controls (Fig. 7).

**Figure 8:**
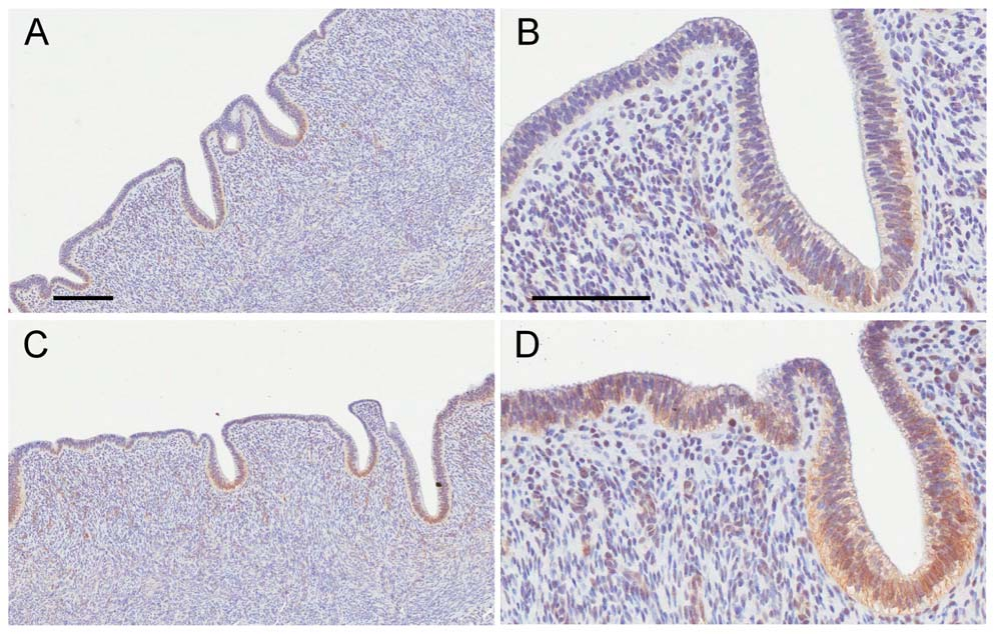
Beta-catenin staining of uteri from control and BPA-treated macaques at GD165. A–B. Control. C–D. BPA-treated. Scale bars = 100 µm.

The overall increases in expression of Wnt signaling-related mRNAs in BPA-treated macaques at GD165 suggested that canonical Wnt signaling via beta-catenin might be induced to a greater extent than in controls. Immunohistochemical staining of GD165 control uteri revealed localized beta-catenin staining at the tip of the developing gland structures (Fig. 8A, B). Uteri from BPA-treated macaques at GD165 appeared to have more intense beta-catenin staining at this location but also exhibited nuclear beta-catenin staining in cells not within glandular structures. However, because of the uneven staining distribution and the different numbers of gland structures across different sections, we were unable to effectively quantify the differences in beta-catenin staining using computer morphometry.

## Discussion

Here we utilized a rhesus macaque model to characterize histology and gene expression in fetal uteri at mid and late gestation and examined the effects of daily *in utero* exposure to BPA on uterine development. In control animals at GD165 there was increased evidence of uterine glandulogenesis on histology and extensive changes in the gene expression profiles compared to GD100. BPA exposure did not result in any significant changes in fetal uterine histology at either GD100 or GD165; however, there were significant differences in gene expression in response to BPA in the GD165 group, particularly in *HOX* and *WNT* family genes.

As in humans, major organogenesis occurs in macaques during the first trimester of pregnancy, and is complete by about GD46 [19]. The BPA exposures in this study were not during the first trimester and thus were not timed to determine effects on major organogenesis. Instead, BPA was given during the more subtle tissue differentiation events of female meiosis and ovarian follicle formation, which occur during the second and third trimesters, respectively [12]. As a result, the BPA was given during gestational periods when little overt morphologic differentiation was occurring in the uterus except for initial glandular morphogenesis in the GD165 group. Exposure to BPA in our study did not significantly alter the appearance of the uterine glands at GD165, suggesting that BPA did not interfere with the epithelial-stromal signaling required to initiate this process.

Evaluation of endometrial cell labeling for ERα and PR, which is induced by ERα activity, revealed that ERα was more highly expressed than PR at both GD100 and GD165. Expression of PR was somewhat higher at GD165 than at GD100, coinciding with a significant increase in expression of *KLF9*, which encodes a modulator of PR function. These findings suggest that there is increased uterine ERα activity and/or PR responsiveness with fetal age. However, no clear differences in ERα or PR-responsive genes were noted between GD100 and GD165 control uteri. The nuclear receptor expression patterns may reflect effects of the relatively high circulating maternal estrogen and progesterone levels during pregnancy, which likely contributed to the presence of subnuclear vacuolization in epithelial cells at both time points (resembling human early luteal phase endometrium). Quantitative analysis of ERα, PR, and Ki-67 staining did not reveal any effects of BPA exposure at GD100 or GD165, indicating that BPA at the dose used did not interfere with expression of these proteins or alter proliferation. Our study design precluded any examination of long-term carryover effects of BPA exposure on these endpoints.

Gene expression analysis of uteri from GD165 showed extensive differences compared to GD100, with major contributions by hematopoietic genes and cell cycle regulators. Although the cell origin of the altered hematopoietic gene expression is unclear, previous studies of *Macaca fascicularis* have reported an increase in the vascular supply to the endometrial stroma between GD100 and GD150 [9]. The pattern of expression of cell cycle regulators was consistent with a decreased uterine growth rate at GD165 as compared to GD100. This finding suggests that although major organogenesis is complete by around GD50, the uterus continues to have actively proliferating cells through GD100 and that this process may slow somewhat in the final trimester. This observation is generally consistent with the pattern of circulating maternal estradiol, which rises rapidly from GD50 to GD80 but then remains at a plateau until the final week before delivery when it rises sharply again [20]. However, because a significant decrease in cell proliferation as indicated by Ki-67 staining was not observed by immunohistochemistry at GD165 (Fig. 2), the impact of these cell cycle-related gene expression changes is uncertain.

Three of the differentially regulated genes associated with BPA exposure in late gestation, *HOXA13*, *WNT4*, and *WNT5A*, are important for urogenital tract development and function in humans [21], [22], [23]. *HOX* genes encode transcription factors that establish the body plan and regulate development during embryogenesis, whereas *WNT* genes encode secreted signaling factors that also regulate development, sometimes in cooperation with HOX genes [24], [25], [26]. Female reproductive tract anterior-to-posterior differentiation is driven by differential expression of the posterior *HOXA* genes, including *HOXA9*, *HOXA10/A11*, and *HOXA13*, which are most highly expressed in the oviduct, the uterus, and the cervix/upper vagina, respectively [27]. The 3-fold decreased expression of *HOXA13* observed here in BPA-exposed animals is of particular note because function-disrupting mutations in this gene cause Hand-Foot-Uterus syndrome, which is characterized by dysplasia of hands and feet along with genitourinary abnormalities in both females and males [23], [28]. *HOXA13* repression, if it occurred during uterine organogenesis, would logically promote similar abnormalities.

Function-disrupting mutations of *WNT4*, a gene critical for female sex determination and organogenesis, are associated with Müllerian aplasia, ovarian follicle depletion, and hyperandrogenism in women [21]. Disruption of both *WNT4* alleles results in numerous developmental defects, including kidney and lung dysgenesis and female to male sex reversal [29]. Localization of beta-catenin to the tips of the developing endometrial glands suggests an important role for canonical WNT signaling in endometrial tubulogenesis, similar to its documented role in tubulogenesis during kidney and lung development [30]. WNT4 protein is detected in human fetal oocytes and granulosa cells throughout gestation and, based on the human *WNT4* mutant ovary phenotype and the *Wnt4*-null mouse model, likely regulates oocyte survival, formation of germ cell cysts, and meiosis [31], [32]. Several *Wnt* genes, including *Wnt4*, are also important for mammary gland development downstream of steroid hormone signals in the mouse (reviewed in [33]). *Wnt4* itself appears to mediate mammary ductal side branching during mouse pregnancy [34]. Our finding that *WNT4* was upregulated in the BPA-exposed fetal uterus is consistent with our identification of progesterone signaling as an upstream regulator of the differentially expressed genes (Fig. 7). It is unclear at this point whether BPA-induced alterations in *WNT4* expression could occur in the ovary or mammary gland and thereby contribute to the abnormalities in follicle formation and mammary gland budding observed at birth in offspring of BPA-exposed macaques [12], [13].

In both mice and women, *WNT* and *HOX* gene expression persists in the female reproductive tract and other tissues past embryogenesis and into adulthood [27], [35], [36], [37]. These findings suggest ongoing roles for these genes in human adult tissues, including regulation of endometrial function [27], [38]. In the mouse, *Wnt4* is upregulated by estradiol, expressed in peri-implantation decidua, and required for uterine gland formation and proper adult uterine function [37], [39]. *Wnt5a* mediates stromal-epithelial interactions during uterine gland formation in mouse neonates [26]. In humans, uterine gland formation is a prolonged process that begins *in utero* but continues to occur through adolescence based on the increasing extent of uterine glands observed through puberty [40], suggesting that disruption of *WNT* and/or *HOX* expression postnatally in girls could alter gland formation. Prenatal BPA exposure can permanently modify expression of ERα and PR in mouse endometrium [41] and result in persistent epigenetic alterations that affect estrogen-mediated transcriptional responses such as upregulation of *Hoxa10* expression [42], [43]. These findings, combined with evidence here that BPA may disrupt expression of multiple *HOX* and *WNT* genes in the fetal primate uterus, suggest that the full impact of prenatal BPA exposure on the uterus may not be evident until later in life. Studies designed to answer this question will require long-term followup of exposed animals into adulthood.

## Supporting Information

Table S1 Microarray gene expression validation
(PDF)

Table S2 Selected biological function categories identified by Ingenuity analysis in comparison of control GD100 vs. GD165 animals
(PDF)

Table S3 Selected upstream regulators identified by Ingenuity analysis in comparison of control GD100 vs. GD165 animals
(PDF)

Table S4 Selected upstream regulators identified by Ingenuity analysis in comparison of GD165 control vs. BPA treated animals
(PDF)
