# Supplementary material for: Bisphenol A Exposure Alters Developmental Gene Expression in the Fetal Rhesus Macaque Uterus
Source: PLoS One. 2014 Jan 23;9(1):e85894. doi: 10.1371/journal.pone.0085894 (PMC3900442; doi:10.1371/journal.pone.0085894)
Supplement: Table S1 — Microarray gene expression validation (PDF) [file pone.0085894.s001.pdf]

Table S1. Microarray gene expression validation

Gene	Primers (5'-3')[a]	Microarray Fold-Change (GD165/GD100)	PCR Fold-Change (GD165/GD100)
HBB	F:CTGCATGTGGATCCTGAGAA<br>R:CCCCCAGTTCAGTAGTTGGA	87.45	246.15
ZBTB16	F:TCTGTCTGCTGTGTGGGAAG<br>R:TTTGTGGCTCTTGAGTGTGC	18.83	14.16
KLF9	F:ACAGTGGCTGTGGGAAAGTC<br>R:AACTGCTTTTCCCCAGTGTG	18.20	22.58
NNMT	F:TGGCCCCACTATCTATCAGC<br>R:CACGTCACACTTCAGCACCT	15.85	10.28
WIF1	F:ATTGCTCAACCACCTGCTTT<br>R:CCCTGGTAACCTTTGGAACA	5.06	3.26
NKAIN4	F:CTGTCTGCACGAGGAGGTG<br>R:CGTAGCAGCCACAGACAAAA	-2.86	-7.69
GAS2	F:CTTGCAGAAACTGTGCAGGA<br>R:TCATCCACCCCTAAATCTCG	-3.70	-2.70
FBN3	F:GAACCCCAGTGTTTGTGACC<br>R:AAAGGAACCCCTCGTGTTCT	-4.55	-6.25
SULT1E1	F:TGCCACCTGAACTTCTTCCT<br>R:TCCTTGCATGAATTTCTCCAC	-5.88	-5.88
LIX1	F:GCACTTTAGATGATGCGGATG<br>R:ACGAAGGGCTTTTAGGCTTC	-6.25	-5.56
**Gene**	**Primers (5'-3')[a]**	**Microarray Fold-Change (GD165 BPA/Control)**	**PCR Fold-Change (GD165 BPA/Control)**
HOXC9	F:GCAGCAAGCACAAAGAGGAG<br>R:CTTCTCCAGTTCCAGCGTCT	2.51	2.47
WNT2	F:CTGTATCAGGGACCGAGAGG<br>R:TGACATGGGAGGTGTCGTAG	1.99	2.16
WNT4	F:CCCTCATGAACCTCCACAAC<br>R:ACCTCACAGGAGCCTGACAC	1.56	1.46
HOXA13	F:CCTCTGGAAGTCCACTCTGC<br>R:ATTTGTTCGTGGCGTATTCC	-3.31	-11.25
SST	F:CCCAGACTCCGTCAGTTTCT<br>R:ATCATTCTCCGTCTGGTTGG	-9.05	-13.05

[a]F, forward; R, reverse
