# Supplementary material for: Bisphenol A Exposure Alters Developmental Gene Expression in the Fetal Rhesus Macaque Uterus
Source: PLoS One. 2014 Jan 23;9(1):e85894. doi: 10.1371/journal.pone.0085894 (PMC3900442; doi:10.1371/journal.pone.0085894)
Supplement: Table S2 — Selected biological function categories identified by Ingenuity analysis in comparison of control GD100 vs. GD165 animals (PDF) [file pone.0085894.s002.pdf]

Table S2. Selected biological function categories identified by Ingenuity analysis in comparison of control GD100 vs. GD165 animals[a]

Category	Functions Annotations	Activation z-score range	Maximum # molecules
Cell Cycle	S phase, segregation of chromosomes, interphase of tumor cell lines	-2.35 to -2.04	60
Cellular Assembly and Organization	Association of chromosome components, segregation of chromosomes	-2.79 to -2.35	33
Cell Death and Survival	Cytotoxicity, cell death of phagocytes, cytotoxicity of leukocytes and lymphocytes, cell death of antigen presenting cells	2.34 to 3.68	44
Cell-To-Cell Signaling and Interaction	Activation of cells, blood cells, leukocytes; recruitment of cells, blood cells, leukocytes, phagocytes, myeloid cells, granulocytes, neutrophils; response of mononuclear leukocytes; activation of connective tissue cells; sensitization of antigen presenting cells, phagocytes	2.00 to 3.58	153
Cellular Development	Differentiation of leukocytes, lymphocytes; maturation and expansion of blood cells, leukocytes; proliferation of stromal cells	2.05 to 3.34	106
Cellular Function and Maintenance	Function of blood cells, leukocytes, antigen presenting cells, phagocytes; cellular homeostasis; T cell homeostasis	2.01 to 3.13	207
Cellular Movement	Cell movement; cell movement of blood cells, leukocytes, phagocytes, myeloid cells, granulocytes, lymphocytes, antigen presenting cells, neutrophils; migration of cells, leukocytes; recruitment of blood cells, phagocytes, myeloid cells, granulocytes, neutrophils	2.12 to 4.12	324
Hematological System Development and Function	Accumulation of leukocytes; activation of blood cells, leukocytes; cell movement of antigen presenting cells, granulocytes, leukocytes, lymphocytes, myeloid cells, neutrophils, phagocytes; differentiation of leukocytes, lymphocytes; expansion of leukocytes, T lymphocytes; quantity of blood cells, leukocytes, lymphocytes; recruitment of granulocytes, leukocytes, myeloid cells, neutrophils, phagocytes; T cell homeostasis	2.13 to 4.09	172
Inflammatory Response	Accumulation and activation of leukocytes; cell movement of neutrophils, phagocytes; cell-mediated response; chemotaxis of granulocytes, leukocytes, phagocytes; inflammatory response; recruitment of neutrophils, phagocytes	2.13 to 4.09	120
Tissue Morphology	Quantity of blood cells, leukocytes, lymphocytes, mononuclear leukocytes, T lymphocytes	2.19 to 3.50	172
Lipid Metabolism	Fatty acid metabolism; synthesis of fatty acid, eicosanoid, prostaglandin E2; metabolism of eicosanoid	2.49 to 3.05	87

[a]Selected from Ingenuity biological functions with predicted activation state significantly increased or decreased as indicated by an activation z-score > 2.0 or < -2.0; all p-values < 0.001.
