# Supplementary material for: Bisphenol A Exposure Alters Developmental Gene Expression in the Fetal Rhesus Macaque Uterus
Source: PLoS One. 2014 Jan 23;9(1):e85894. doi: 10.1371/journal.pone.0085894 (PMC3900442; doi:10.1371/journal.pone.0085894)
Supplement: Table S3 — Selected upstream regulators identified by Ingenuity analysis in comparison of control GD100 vs. GD165 animals (PDF) [file pone.0085894.s003.pdf]

Table S3. Selected upstream regulators identified by Ingenuity analysis in comparison of control GD100 vs. GD165 animals[a]

Upstream regulator	Description	Activation z-score	p-value of overlap	Mechanistic network
TBX2	T-box 2; transcription regulator	-3.99	2.59E-08	Ap1,CDKN1A,E2f,RB1,Rb,TBX2,TP53
EP400	E1A binding protein p400	-3.64	3.40E-16	E2F2,EP400,Histone h4
FOXM1	forkhead box M1; transcription regulator	-3.51	5.47E-09	Ap1,CDKN1A,E2F1,E2f,FOXM1,HDAC1,NFATC2, RB1,Rb,TP53,Vegf
CTNNB1	catenin (cadherin-associated protein), beta 1	-3.10	5.53E-03	ATF2,Ap1,CTNNB1,FOS,JUN,JUND,SP1,TCF
CCND1	cyclin D1	-2.61	5.29E-20	Ap1,CCND1,CCNE1,CDKN1A,E2F1,E2F2,E2F3, E2F4,E2F5,E2f,HDAC1,RB1,RBL1,Rb,TP53
E2F3	E2F transcription factor 3; transcription regulator	-2.58	5.38E-07	BRCA1,CCNE1,E2F1,E2F3,E2F4,E2F5,E2f, HDAC1,RB1,RBL1,TP53
E2F1	E2F transcription factor 1; transcription regulator	-2.56	5.19E-06	CCNE1,E2F1,E2F2,E2F3,E2F4,E2F5,E2f,HDAC1, RB1,RBL1,Rb,TBX2,TP53
mir-21	microRNA	-2.54	4.48E-03	IFNG,IRF1,NFKB1,NFkB(complex),STAT1,STAT3, mir-21
E2F2	E2F transcription factor 2; transcription regulator	-2.24	3.56E-06	CCNE1,E2F1,E2F2,E2F4,E2f,HDAC1,RB1,TP53
estrogen	steroid hormone	-2.22	6.47E-05	1-alpha,25-dihydroxy vitamin D3,Ap1,CEBPB, CTNNB1,E2F1,E2F2,E2F3,E2F4,E2f,ESR1,HDAC1, IL6,JUN,JUNB,NCOA2,NRIP1,RB1,Rb,SP1,STAT3, TCF3,TP53,VDR,estrogen
TNF	tumor necrosis factor; cytokine	2.98	3.84E-08	CEBPB,E2F4,IFNG,IL10,IL1B,IL6,IRF1,IRF3, JUN,JUNB,NFKB1,NFKBIA,NFkB(complex), RELB,STAT1,STAT3,TCF3,TNF,TP53
IFNA2	interferon, alpha 2; cytokine	3.04	4.90E-05	BAX,CREBBP,IFNA2,IL12(complex),IRF1,IRF3, IRF9,JUN,NFKB1,NFkB(complex),STAT1,STAT2, STAT3,TP53,TRIM21
CEBPA	CCAAT/enhancer binding protein (C/EBP), alpha; transcription regulator	3.10	4.39E-03	AR,ATF2,Ap1,CEBPA,CEBPB,CREB1,CREBBP, E2F1,FOS,HDAC1,JUN,JUND,MYOD1,RB1, SMARCA4,SP1,STAT1,STAT3,TP53
STAT1	signal transducer and activator of transcription 1; transcription regulator	3.10	6.10E-04	CREBBP,IFNG,IL10,IL12(complex),IRF1,IRF3, IRF9,NFKB1,NFkB(complex),STAT1,STAT2, STAT3
RB1	retinoblastoma 1; transcription regulator	3.12	4.06E-07	BRCA1,CCNE1,E2F1,E2F2,E2F3,E2F4,E2F5,E2f, HDAC1,RB1,RBL1,RBL2,Rb,TP53,TP73
SMARCB1	SWI/SNF related, matrix associated, actin dependent regulator of chromatin b1, transcription regulator	3.20	5.21E-04	Ap1,BRCA1,CDKN1A,CDKN2A,E2F1,E2F4,E2f, HDAC1,MYOD1,RB1,Rb,SMARCA4,SMARCB1, STAT3,TP53
JUN	jun proto-oncogene; transcription regulator	3.58	6.07E-04	ATF2,Ap1,CEBPB,E2F4,ESR1,FOS,IL6,JUN, JUNB,JUND,NFATC2,SP1,STAT3,TCF3
IFNG	Interferon, gamma; cytokine	3.66	5.01E-06	CEBPB,CREBBP,IFNG,IL10,IL1B,IRF1,IRF3, IRF9,JUN,NFKB1,NFKBIA,NFkB(complex), SMARCA4,STAT1,STAT2,STAT3,TNF,TP53
TP53	tumor protein p53; transcription regulator	4.17	3.03E-11	Ap1,CDKN1A,E2F1,E2F4,E2F5,E2f,ERBB2,FOS, HDAC1,RB1,RBL2,Rb,SP1,STAT3,TBX2,TP53
let-7	microRNA	4.44	2.13E-09	

[a]List filtered to keep only the upstream regulators with an activation z-score > 2.0 or < -2.0, ≥ 10 target molecules, and p-value of overlap < 0.01.
