# Supplementary material for: Bisphenol A Exposure Alters Developmental Gene Expression in the Fetal Rhesus Macaque Uterus
Source: PLoS One. 2014 Jan 23;9(1):e85894. doi: 10.1371/journal.pone.0085894 (PMC3900442; doi:10.1371/journal.pone.0085894)
Supplement: Table S4 — Selected upstream regulators identified by Ingenuity analysis in comparison of GD165 control vs. BPA treated animals (PDF) [file pone.0085894.s004.pdf]

Table S4. Selected upstream regulators identified by Ingenuity analysis in comparison of GD165 control vs. BPA treated animals[a]

Upstream regulator	Description	Activation z-score	p-value of overlap	# target molecules	Mechanistic network
mifepristone	progesterone receptor antagonist	-1.88	4.13E-04	13	PGR, mifepristone, progesterone
progesterone	steroid hormone	1.53	1.59E-03	17	PGR, progesterone
AR	androgen receptor	1.53	4.50E-03	11	
dihydro-testosterone	steroid hormone	1.69	3.24E-02	14	
rosiglitazone	thiazolidinedione; insulin sensitizer	1.85	5.53E-03	13	
IFNAR	interferon-α/β receptor	1.93	1.35E-03	4	
PKD1	polycystin 1; ion channel	1.94	1.83E-02	7	
anisomycin	antibiotic	1.95	4.21E-03	4	
MLL	mixed lineage leukemia gene; transcription regulator	1.98	1.05E-03	4	
STAT1	Signal transducer and activator of transcription 1; transcription regulator	2.22	3.90E-02	8	

[a]List filtered to keep upstream regulators with an activation z-score > 1.5 or < -1.5 and p-value of overlap < 0.05.
